# High-throughput sequencing data and the impact of plant gene annotation quality

**DOI:** 10.1093/jxb/ery434

**Published:** 2018-12-24

**Authors:** Aleksia Vaattovaara, Johanna Leppälä, Jarkko Salojärvi, Michael Wrzaczek

**Affiliations:** 1 Organismal and Evolutionary Biology Research Programme, Viikki Plant Science Centre, VIPS, Faculty of Biological and Environmental Sciences, University of Helsinki, Viikinkaari 1 (POB65), Helsinki, Finland; 2 Department of Ecology and Environmental Science, Umeå University, Linnaeus väg 6, Umeå, Sweden; 3 School of Biological Sciences, Nanyang Technological University, Singapore, Singapore

**Keywords:** Gene families, genome annotation, GWAS, high-throughput sequencing, phylogeny, translational research

## Abstract

The use of draft genomes of different species and re-sequencing of accessions and populations are now common tools for plant biology research. The *de novo* assembled draft genomes make it possible to identify pivotal divergence points in the plant lineage and provide an opportunity to investigate the genomic basis and timing of biological innovations by inferring orthologs between species. Furthermore, re-sequencing facilitates the mapping and subsequent molecular characterization of causative loci for traits, such as those for plant stress tolerance and development. In both cases high-quality gene annotation—the identification of protein-coding regions, gene promoters, and 5′- and 3′-untranslated regions—is critical for investigation of gene function. Annotations are constantly improving but automated gene annotations still require manual curation and experimental validation. This is particularly important for genes with large introns, genes located in regions rich with transposable elements or repeats, large gene families, and segmentally duplicated genes. In this opinion paper, we highlight the impact of annotation quality on evolutionary analyses, genome-wide association studies, and the identification of orthologous genes in plants. Furthermore, we predict that incorporating accurate information from manual curation into databases will dramatically improve the performance of automated gene predictors.

## Introduction

The ongoing development of next-generation sequencing techniques has led to a remarkable decrease in the cost of genome sequencing. This is reflected in the increasing number of genome assemblies from all domains of life. For plants, more than 230 angiosperm genomes are currently available (Chen *et al.*, 2018). The advances in sequencing technology have also rapidly improved the quality, throughput, and the length of the reads. The result has been a dramatic increase in the amount and quality of information available for biological research, which often relies on gene model annotations representing exon–intron structures (including alternatively spliced isoforms), regulatory elements [e.g. promoter elements, enhancers, as well as 5′- and 3′-untranslated regions (UTRs)], and locations of transposable elements (TEs) and repeat sequences. Therefore, high-quality genome annotations are essential for analyses relying on genomic data. Since a large proportion of the genome is constituted of repetitive sequences and transposable elements varying, e.g., from 15% of the *Arabidopsis thaliana* genome (de la Chaux *et al.*, 2012) up to 85% in maize (Schnable *et al.*, 2009) and bread wheat (Wicker *et al.*, 2018), genome assembly and annotation can be challenging. Errors in gene annotation have a strong impact on the results obtained, especially in phylogenomic analyses or in the functional interpretation of single-nucleotide polymorphisms detected in genome-wide association studies. These effects are more pronounced among tandemly duplicated genes and large gene families. However, the effects of erroneous gene annotations are still overlooked in many studies.

## The importance of high-quality genome data

After the completion of a draft genome sequence, a standard approach is to first identify TEs and other repetitive DNA sequences and then to mask these parts to facilitate the prediction of protein-coding genes from the rest of the genome. A majority of TEs are located in heterochromatic regions but they can also be found in close proximity of genes. For example, in maize 33% of genes have TE insertions in introns (Schnable *et al.*, 2009) and 15% of Arabidopsis genes have TE insertions located within 500 bp from the 3′ or 5′ end of the coding region (Hollister *et al.*, 2011). This may hamper gene annotation, as parts of the genes (in this case intron sequences) could be masked due to repeat annotation. Alternatively, repeat masking that is too cautious can result in false positive gene predictions and produce inflated numbers of predicted genes. These in turn can cause problems in estimating the size of gene families, since TEs often contain fragments of functional genes. Therefore, it is important to develop ways to measure the accuracy of TE identification and annotation, a subject that has recently received attention (Hoen *et al.*, 2015). Gene annotation is typically initiated by so-called *de novo* gene prediction software such as Glimmer (Delcher *et al.*, 1999), SNAP (Korf, 2004), Augustus (Stanke *et al.*, 2008), EuGene (Foissac *et al.*, 2008), Genemark (Ter-Hovhannisyan *et al.*, 2008), or BRAKER (Hoff *et al.*, 2016). The programs learn a statistical model that predicts gene models from genome sequences. The model parameters are trained using evidence from RNA-sequencing data, expressed sequence tags (ESTs), annotated gene models in related species, or by using predictor parameters optimized for a model species. These automated predictions are then typically combined with evidence from RNA-sequencing and known gene models from other sources, for example from related species, using combiner software packages such as EVidenceModeler (Haas *et al.*, 2008), JIGSAW (Allen and Salzberg, 2005), or MAKER (Cantarel *et al.*, 2008). Proteogenomics have also been utilized to improve gene annotations in plants (Castellana *et al.*, 2008; Chapman and Bellgard, 2017). Several recent genome papers have paid specific attention to gene annotation quality and its improvement, for example in the genomes of kiwifruit (Pilkington *et al.*, 2018), silver birch (Salojärvi *et al.*, 2017), wheat (International Wheat Genome Sequencing Consortium, 2018), and melon (Ruggieri *et al.*, 2018). The kiwifruit and the silver birch publications in particular have highlighted the importance of manual curation of automatically predicted gene models.

The quality of a gene annotation is typically assessed through the presence and correctness of well-known single-copy genes (a set of 1440 in plants) called ‘Benchmarking Universal Single-Copy Orthologs’ (BUSCO; Simão *et al.*, 2015) or using a smaller set of genes in the ‘Core Eukaryotic Genes Mapping Approach’ (CEGMA) (Parra *et al.*, 2007). The BUSCO scores for well-annotated genomes vary between 95–97%, with 3–5% of missing or fragmented gene annotations (Raymond *et al.*, 2018; Springer *et al.*, 2018). However, conserved single-copy genes do not necessarily provide a suitable indicator for annotation quality for genes that have duplicates, such as members of gene families. This is particularly relevant for plant genomes where over 80% of genes belong to gene families (Guo, 2013). To evaluate gene family annotations, ‘core gene families’ (coreGFs) (Li *et al.*, 2016; Veeckman *et al.*, 2016) have been proposed in order to provide a measure for the presence of conserved gene families in a genome. Plants have experienced several whole-genome duplications that, together with local tandem duplications, have contributed to expansions of gene families. Due to their high sequence similarity, recently expanded gene families are highly susceptible to annotation problems and tools such as *DuplicationDetector* (Djedatin *et al.*, 2017) and NLR-Parser (Steuernagel *et al.*, 2015) have been developed to detect and correct problems following initial annotation.

Only a few comparisons of the quality of plant genome assemblies and annotations are currently available. Shangguan *et al.* (2013) assessed the quality of 32 plant genomes by mapping ESTs to genome sequences and found that, at the time of their study, the quality of many plant genomes was lower than had previously been assumed, whilst Veeckman *et al.* (2016) used ESTs, CEGMA genes, BUSCO genes, as well as coreGFs to evaluate the annotation quality of 12 plant genomes. In both studies, well-annotated plant genomes such as the model plant species *Arabidopsis thaliana* and rice (*Oryza sativa*) received high scores for genome and annotation quality. Arabidopsis and rice gene annotations have been heavily curated as a result of continuous input from the scientific community, resulting in constantly improving gene annotations. This shows the importance of constant reannotation and correction of gene models, but importantly also serves as a reminder to be aware of potential annotation errors when analysing the genomes of non-model species. However, even well-curated genomes can still contain annotation errors. For example, the gene models for the receptor-like protein kinases (RLKs) *AtCrk16* and *AtCrk17* (Vaattovaara *et al.*, 2018, Preprint) were found to contain annotation errors in the version TAIR10 (TAIR, 2010) of the *Arabidopsis thaliana* genome (the ectodomain region of *Crk16* was annotated as part of *Crk17* and the gene model for *Crk16* was truncated to contain only the kinase domain). This has been corrected in Araport11 (Cheng *et al.*, 2017), but the old versions are still listed as splice variants. Similarly, the gene model for the protein kinase *AtHt1* was only partially predicted (missing 45 AA from the N terminus) in the TAIR10 annotation for *Arabidopsis thaliana* (Hõrak *et al.*, 2016). More drastic gene annotation errors were identified during re-sequencing of resistance genes from the tomato genome when 317 previously unannotated NB-LRR genes were revealed (Jupe *et al.*, 2013).

The issues leading to incorrect annotations of gene models can be diverse. The causative error types behind incorrect gene model predictions can be categorized into sequencing errors, assembly errors, or difficulties with annotation. Sequencing errors typically occur in regions with low sequencing coverage where errors in individual reads can introduce stop codons or frame shifts, resulting in erroneous gene models. The end result of a genome assembly is typically a large number of scaffolds, assemblies where contiguous genomic sequences (typically referred to as contigs) are linked by gaps filled by ambiguous character (Ns). Assembly errors, meaning the erroneous linking of contigs, may lead to truncated or fused gene models or frame shifts. Gaps in assemblies can also result in partial gene models if they overlap with a gene. Similarly, genes may be located at the edges of scaffolds, causing partial or missing annotations. Similar to assembly errors, annotation errors can lead to erroneous gene models, but there can also be problems in gene structure, such as missing or extra exons. A single gene may be split into several genes, or several genes could be fused into a single-gene model, or the splice sites can be misplaced. In case of gene family members organized in tandem repeats, automated gene predictors can in some cases predict a fused-gene model by combining exons in consecutive genes. Naturally, genes can also be entirely missed by annotation algorithms. Even in ‘simpler’ prokaryotic genomes, where genes generally do not contain introns, unannotated genes pose a problem (Warren *et al.*, 2010).

Information from transcriptome sequencing yields high-quality evidence for genes with high transcript abundance and is therefore an invaluable source for gene annotation. In particular, long introns can cause problems in the annotation of the gene models, as gene prediction programmes may split a single gene into truncated partial-gene models. Evidence from transcript data can be helpful in many such cases. Notably, plant genes can contain very long introns, such as *OsMADS50* with the first intron being 27.6 kb long (Tadege *et al.*, 2003); however, this is very rare. In Arabidopsis less than 1% of introns are longer than 1kb (Chang *et al.*, 2017), whereas the same figure in Norway spruce is 24% (Nystedt *et al.*, 2013). There are limitations to the information that can be obtained from transcripts as it is challenging to obtain a comprehensive set of transcripts for all genes in a genome. Typically, only 60–70% of the genes encoded in a genome are expressed in the sampled material (Salojärvi *et al.*, 2017). A large number of transcripts are differentially regulated in response to circadian rhythms, developmental cues, environmental signals, or stress conditions. In multicellular organisms, genes may also have expression patterns that are highly cell- or tissue-specific, resulting in low abundance of corresponding mRNAs in some tissues. In addition to the biological challenges, *de novo* assembled transcriptome data can contain assembly errors. A reference-guided transcriptome assembly, on the other hand, can suffer from assembly errors in a genome that, when used to support gene prediction, can lead to errors in gene models. Although combiner software can search for stop codons to identify the approximate end of a gene (Haas *et al.*, 2008), partial transcripts can lead to the annotation of truncated gene models in the absence of other evidence. For members of large gene families with high sequence similarities it can be difficult to distinguish splice variants and recently diverged gene models, especially if the transcriptome data is sequenced from a different individual. This problem can be expected to become more prominent in the future as more genomes from (auto)polyploid plants become available. Finally, a complementary source of gene annotation, use of predicted gene models and proteins from other available genomes, can possibly lead to inherited erroneous annotations, as has been observed in case of functional annotations (Gilks *et al.*, 2002). Further support for the correctness of gene models can come from the domain composition of the encoded protein. Identification of partial Pfam domains (https://pfam.xfam.org/) has been found to be an excellent indicator of possible annotation errors in gene models (Triant and Pearson, 2015). Therefore, while partial protein domains can arise through incomplete duplications, the identification of a truncated domain warrants additional validation of the gene annotation.

Correct gene annotation with a high degree of completeness is essential for the functional annotation of the genes (Jones *et al.*, 2007; Schnoes *et al.*, 2009), such as the gene ontology assignment and identification of conserved protein domains, and for all subsequent analyses utilizing this information. Variation in the quality of genome and gene annotations can especially cause problems in comparative and evolutionary analyses. Thus, it is necessary to manually validate the annotation quality of different genomes and data sets extracted from available genomes.

## The importance of accurate phylogenies for translational research

While small annotation errors in gene models do not always drastically alter the results of phylogenetic analyses, partial or absent gene models can result in false tree topologies that hamper the interpretation of gene relationships, especially for translational research. Model organisms such as *Arabidopsis thaliana* are a common choice for investigation of gene or protein function (Davis, 2004). Model organisms may have little commercial or agricultural relevance but have relatively small genomes, a short generation time, and can be propagated easily in the laboratory. However, research on a model organism is often carried out to improve the traits of crop species. Orthologous genes by definition have similar functions in different species, thereby allowing transfer of the functional information. With single-copy genes and small gene families the inference of orthologous gene pairs between species is usually simple, but the situation can be more complicated in the case of large gene families. Genome duplications and tandem duplications result in paralogous genes, which can hamper the identification of orthologs that have the same function (Box 1).

Box 1. **Gene families**

Genes in plant genomes are rarely pure single-copy genes with one-to-one orthologs in different species, but instead belong to gene families, a group of genes with common ancestry. An analysis of eight plant and algal genomes found that 86.4% of genes belonged to gene families (Guo, 2013). Gene families can be part of larger superfamilies, for example the plant receptor-like kinase gene family is part of the larger superfamily of protein kinases. The size of gene families can vary drastically, from small families with only few members to very large families with more than thousand. Notably, the number of members in gene families can vary considerably between different species.

Gene families evolve through duplications, pseudogenization, and gene-loss events (see part A of the figure). Duplicated genes can result from whole-genome duplication (WGD) or triplication (WGT) events, from tandem duplication events, from segmental duplication within or between chromosomes, or from duplications mediated by transposable elements (Panchy *et al.*, 2016). A general hypothesis is that following duplication, the duplicates are under decreased selection pressure, which allows mutations to accumulate. This can lead to distribution of ancestral functions between duplicated genes, sub-functionalization, or acquisition of novel functions compared to the ancestral gene (neofunctionalization; Conant and Wolfe, 2008). Genes can be also turned into pseudogenes as a result of the slow accumulation of deleterious mutations, or they can be lost during chromosomal rearrangements. Under strict selection, new duplicates can be removed from the genome to keep the gene as a single-copy gene.

Gene families constitute a striking challenge for translational research. In large gene families the recognition of orthologs (genes separated by speciation) from paralogs (genes that emerge as the result of duplications within species) between distant species can be difficult or even impossible (see part B of the figure). Tandem duplications in particular can lead to large lineage-specific expansions and thus to lineage-specific genes without orthologous genes outside of that specific taxon. The most common problem in translational studies is the assumption that genes having the highest sequence similarity between species are orthologs. This can be erroneous for large gene families, which may have gained and lost genes at different rates in different lineages. In addition, model species have lineage- or species-specific genes and they may have lost certain genes or even gene families. For these reasons, transferring information from large gene families of model species to crops remains a considerable challenge for translational research.

**Figure:**
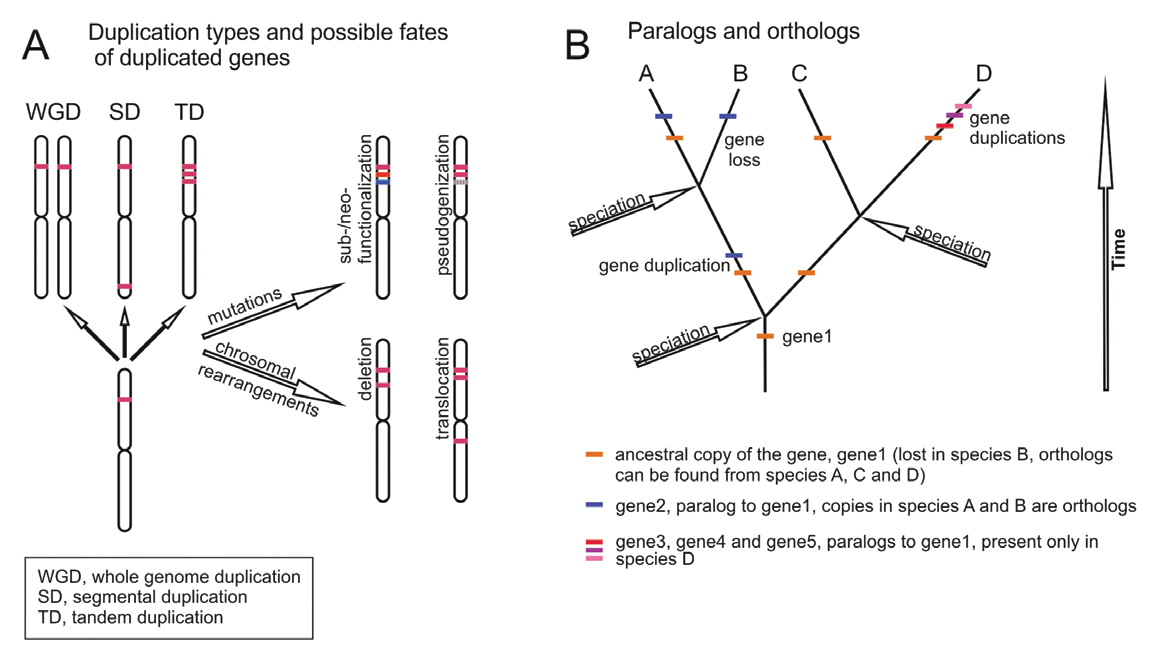

A high-quality phylogenetic tree containing curated gene models from gene family members is an efficient way to investigate relationships among genes from different species (Box 2). However, for large gene families in particular, the high sequence similarity of genes from different species is not necessarily sufficient evidence for orthologs or paralogs, as the similarity-based search does not account for lineage-specific gene duplications and losses. Within gene families, some members may have evolved similarly to conserved single-copy genes, thus facilitating the recognition of orthologs. However, other members may have experienced lineage-specific expansions, thus making the inference of orthologs challenging or even impossible. Synteny, the conservation of the local ordering of a set of genes, provides further evidence of orthology. Finally, transcriptional evidence can be used to determine whether the expression of putative orthologs is conserved, since the altered regulation of gene expression, for example due to changes in the promoter region, is not visible in phylogenetic trees based on the coding region.

Box 2. **Phylogenies**

Phylogenetic trees represent the evolutionary distances between given data samples. In phylogenomics, phylogenies are usually either species trees that represent the relationships between species, or gene (or protein) trees that represent evolutionary relationships between genes and proteins. The distances in the tree are estimated based on the similarities and differences between data samples; for genes and proteins the data are derived from sequence alignments. Nucleotide sequence alignments are informative for closely related sequences, while amino acid alignments can be more practical for more divergent data sets. The construction of phylogenetic trees can be carried out by different methods based on distance matrices, parsimony, maximum-likelihood, or Bayesian methods (Yang and Rannala, 2012). With large data sets, the estimation of the phylogenetic tree is a so-called NP-hard (non-deterministic polynomial-time) problem where there exists a single, correct answer, but it is exponentially hard to identify, and therefore the result represents the optimum from among the trees explored by the search algorithm. The reliability of the splits represented by the nodes of the tree is commonly evaluated by bootstrapping (Felsenstein, 1985; Holder and Lewis, 2003).

Annotation errors can cause severe problems for the inference of phylogenetic trees. Short stretches of missing or extra sequences may only affect the branch lengths in the tree, but missing or additional exons can lead to long branches or even differences in the branching order in a phylogeny. Missing gene models on the other hand can lead to false estimations of the relationships between genes. In part (A) of the figure, which represents members of a gene family from three different species indicated by the letters A–C, two examples of possible annotation errors in the phylogenetic tree are presented. The branch leading to gene B2 is long compared to the branches leading to the other genes in the phylogeny. This indicates either diversifying evolution or that there are problems with the annotation. In the case of gene A6, an annotation error is even more likely, as the gene is grouped with the genes from species C.

The relationships between genes from different species can be defined from comprehensive phylogenies. This is useful for identifying orthologs and paralogs in gene families, for example for translational research where information on gene or protein function in a model species is used to improve crop performance. The recognition of lineage-specific gene expansions and contractions from the phylogeny is important for correct interpretation of the relationships of gene family members between species. In part (B) of the figure, which again represents members of a gene family from three different species, the ancestral gene has duplicated prior to the separation of the species, giving rise to two related clades. In both clades a single gene from species A is placed closest to the root of the tree. In Clade 1, gene B1 is orthologous to A1, while in the lineage leading to species C, gene duplication has taken place, making ortholog identification more difficult. In Clade 2, both species B and C have undergone several lineage-specific gene duplication events. For species B, the inference of orthologous genes between species is not possible based only on the phylogeny. For species C, the gene C3 could possibly be the ortholog for A2 as they are the most similar genes between these species, but sub-functionalization between these genes is also possible.

**Figure:**
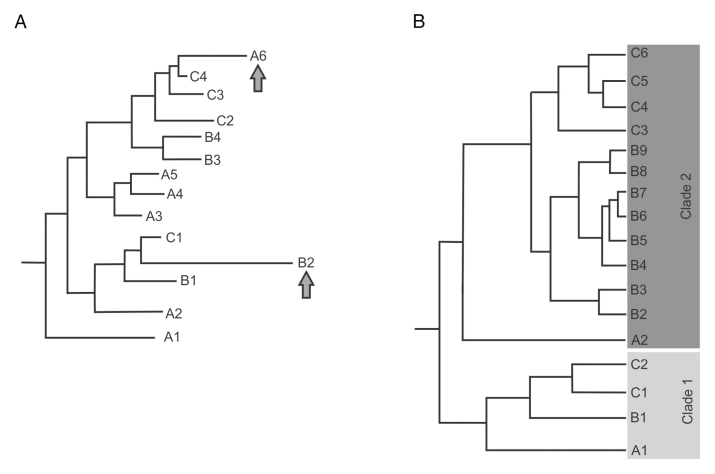

## Genome-wide association studies benefit from high-quality annotations

Identifying causal variants for quantitative traits in model plants and crops is essential for improving agronomically important traits and fundamental for finding adaptive variants in evolutionary biology. High-throughput genome sequencing enables this by providing information on single-nucleotide polymorphisms (SNPs) that can be analysed in a genome-wide association study (GWAS). GWAS utilizes naturally occurring phenotypic variation within populations or species and identifies statistically associated genotypic variation (for a recent review in plants see Ogura and Busch, 2015).

Interpretation of results from GWAS analyses can be challenging. The genetic architecture underlying many common traits is frequently complex (that is, polygenic) and therefore the sizes of the effects of the associated SNPs that have been identified by the GWAS are often small (Ingvarsson and Street, 2011; Visscher *et al.*, 2017), although the proportion of the variation in the trait explained by significant SNPs is generally found to be larger in plants (Huang *et al.*, 2010; Li *et al.*, 2010; Ingvarsson and Street, 2011). Thus, it can be challenging to distinguish true ‘small effect’ associations from artefacts. In addition, several studies have shown that the underlying genetics for adaptive traits can cause significant associations to be thousands of base pairs away from the causative locus, due to multiple alleles being present at a locus and recent positive selection (causing positively associated SNPs to be spread over longer regions due to linkage disequilibrium; e.g. Atwell *et al.*, 2010; Kerdaffrec *et al.*, 2016). Thorough fine-mapping of quantitative trait loci is highly time-consuming, and so high-quality annotation information is essential for determining the location of candidate causative SNPs for validation: SNPs may be located either in protein-coding regions where they can result in synonymous or non-synonymous substitutions, or alternatively in introns, UTRs, or intergenic regions where they possibly have a lower impact on function. Therefore, high-quality structural and functional gene annotations are essential for predicting the likely candidate genes or genomic regions and nucleotide variant(s) that underlie an investigated trait. GWASs frequently concentrate on SNPs located in protein-coding genes. Non-synonymous SNPs can be identified from high-quality annotation data, and prior information, including gene expression data and computational methods, can be used to predict the effect on protein function (Tang and Thomas, 2016). For example, the weeping phenotype of the silver birch garden cultivar ‘Youngii’ was predicted to result from a premature stop codon detected in the *LAZY* gene, known to result in a similar relaxed phenotype in maize, rice, Arabidopsis, and peach (Salojärvi *et al.*, 2017). However, SNP variants outside coding regions can also be functional, for example by affecting regulation of gene expression or transcript stability. In addition, transposable elements can affect the expression of nearby genes, resulting in changes in the plant phenotype (reviewed by Cui and Cao, 2014). It is therefore crucial to improve the annotation and functional understanding of the non-coding parts of genomes, which typically comprise the majority of the genome. Recent studies have identified long non-coding RNAs (Liu *et al.*, 2015), open chromatin regions (Rodgers-Melnick *et al.*, 2016), small open reading frames (ORFs; Hellens *et al.*, 2016), epigenomes (Kawakatsu *et al.*, 2016), and transcription factor binding sites (cistromes; O’Malley *et al.*, 2016). It would be valuable to obtain such information from many different tissue and cell types. Increased knowledge of the functional roles of non-coding regions will greatly improve identification of putative causal SNPs from GWASs. In human genetics, tools to predict the functions of non-coding SNPs have been developed (reviewed by Nishizaki and Boyle, 2017) and hopefully similar tools will soon become available for plant model species, and subsequently more widely for non-model species.

Similar to phylogenetic approaches, GWASs are also strongly affected by the quality of genome assemblies. When high-throughput sequencing is used for genotyping, the SNPs are typically identified against a reference genome. A genome sequence that is absent from the reference but present in some individuals is usually excluded in standard analyses. If the size of the excluded insert is large and it has a causative variant, the remaining associated SNPs may be too far away and thus the size of their effect may be too low to be detected. Functional interpretation is therefore not possible because the variants, which potentially have a causative effect, are not included in the analysis.

Using additional resources such as gene-expression and protein-interaction networks together with high-quality genome annotation will in the future result in improved understanding of GWAS results, and ultimately in improved understanding of the genetic architecture of different traits. Careful reannotation of regions containing significant SNPs is strongly advisable to verify their location in coding or non-coding regions. In the future, GWASs will benefit from integration of improved, curated annotations of both protein coding and non-coding regions into the available reference genome information.

## Conclusions

Over the past decade, high-throughput sequencing has improved significantly and genome data for different plant species, accessions, and populations are now widely available. Reference genomes can be used as a tool for evolutionary analyses, for the mapping and characterization of agriculturally important traits, and for translational research. However, despite the increasing number and quality of available genomes, the quality of their assembly and annotation is variable, and in some cases this can represent a serious problem for detailed analyses.

Overall, as sequencing technology develops, assemblies become more contiguous and contain fewer assembly errors. At the same time, gene annotation software is developing rapidly and is already able to overcome commonly observed annotation problems, whilst RNAseq is able to provide reliable evidence for gene model structures. The development of combiner software, which is able to make composite gene predictions based on several data sources, has been a major recent advance in gene annotation, and our view is that it should be the main focus when aiming to further improve annotation quality, perhaps by including more diverse information sources such as phylogenomics, synteny, and information on tandem expansions.

For the time being, careful validation and *de novo* annotation of gene models is particularly important not only for members of large gene families, but also for genes where transcriptional evidence is difficult to obtain, such as those transcribed in response to specific environmental stimuli or that have high tissue specificity. Manual curation of annotations allows the identification of orthologous genes with higher confidence for translational research, and also strengthens the GWAS approach for the identification of causative loci for traits of high importance to agriculture.

In spite of the increasing quality of gene and genome annotations, we want to emphasize that the careful reannotation of genomic regions of interest remains an important tool for analyses of gene families, for translational approaches, and also for GWASs and mutant screens. The procedure should consist of reannotation of TEs, ORFs, and exon–intron structures, as well as promoter and UTR regions. Encoded proteins should be checked for the completeness of Pfam domains and for whether they correspond to the typical domain architecture of related proteins. Making these curated annotations available in genome databases will dramatically increase the quality of genomes and gene annotations. More importantly, more accurate information in databases makes it possible to improve automated gene predictors, which in turn will reduce the effort required for manual reannotation of genomic features.
